# Optimization of milling operation parameters using Python-assisted GRA for enhanced surface quality and material behaviour

**DOI:** 10.1039/d6ra03051c

**Published:** 2026-07-30

**Authors:** Ganeshkumar Selvaraj, Ponni Ponnusamy, Ramakrishnan Thirumalaisamy, Manickaraj Karuppusamy, Jenyfal Sampson, Pravin Manikandan, Nivin Joy, Karthikayan Sundararajan, Kuwar Mausam, Sivasubramanian Palanisamy, Aravindhan Alagarsamy, Mezigebu Belay

**Affiliations:** a Department of Mechanical Engineering, Sri Eshwar College of Engineering Coimbatore – 641202 Tamilnadu India; b Department of Computer Science and Business Systems, Sri Eshwar College of Engineering Coimbatore – 641202 Tamil Nadu India; c Department of Mechanical Engineering, CMS College of Engineering and Technology Coimbatore – 641032 Tamilnadu India; d Department of Electronics and Communication Engineering, Kalasalingam Academy of Research and Education Krishnankovil – 626126 Tamilnadu India; e Department of Information Technology, CMS College of Engineering and Technology Coimbatore – 641032 Tamilnadu India; f Department of Mechanical Engineering, Sathyabama Institute of Science and Technology Chennai Tamilnadu India; g Department of Mechanical Engineering, School of Engineering, Mohan Babu University Tirupati Andhra Pradesh – 517102 India; h Department of Mechanical Engineering, GLA University Mathura India; i Center for Excellence Flexible Electronics, Department of Electronics and Communication Engineering, Koneru Lakshmaiah Education Foundation Vaddeswaram Andra Pradesh 522501 India; j Department of Metallurgical and Materials Engineering, College of Engineering, Ethiopian Defence University Bishoftu 1041 Ethiopia sivaresearch948@gmail.com mezgebubelay@etdu.edu.et

## Abstract

Modern manufacturing's paradigm has changed a lot with the inclusion of optimization and smart manufacturing techniques. In this work, the performance of milling operation of magnesium alloy (Mg-AZ61) has been studied by using Box–Behnken Design (BBD) with Grey Relational Analysis (GRA). The main aim of this work is to optimize the operating parameters for better surface quality. In the milling experiments, important key process parameters namely spindle speed, axial depth and cutting feed rate were considered. The Box–Behnken experimental design matrix is prepared to conduct the milling trials in a systematic manner. Then GRA is applied to evaluate the relationship between the operating parameters and the surface integrity. The measured data were normalized and the best parameter combination was obtained by GRA. The GRA identified the optimum process parameters within the investigated experimental domain. The optimized operating conditions produced improved surface quality while maintaining satisfactory material removal performance. The obtained results demonstrate the effectiveness of the proposed BBD–GRA framework for multi-response optimization of Mg-AZ61 milling. The proposed BBD–GRA model predicted an optimized surface finish of 0.3168 µm, which was subsequently validated through confirmation experiments. This indicates a substantial, improvement as compared to the non-optimized conditions. Moreover, this paper presents the potential of combining GRA with machine learning models for adaptive and intelligent manufacturing systems.

## Abstract 2

Modern manufacturing's paradigm has changed a lot with the inclusion of optimization and smart manufacturing techniques.

## Graphical abstract

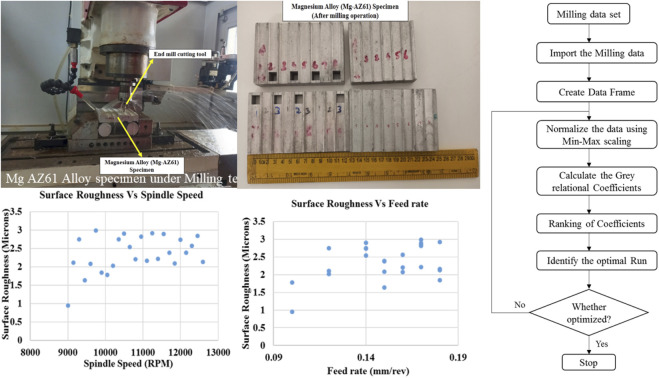

## 1. Introduction

Autonomous and intelligent manufacturing systems have been an integral part of smart manufacturing paradigms in recent years. The rapid development of Industry 4.0 has resulted in the deployment of advanced digital technologies directed toward increasing productivity, process reliability and product quality while reducing manufacturing costs and material waste.^1^ The traditional machining processes that were based on fixed parameter selection and the experience of the operator are gradually changing with the use of optimization techniques and real-time monitoring systems. This transition has allowed for manufacturing systems to be more adaptive, precise, and consistent in various operating conditions.^2^

The integration of traditional optimization methods into real-time machining environments has dramatically enhanced machining performance by enabling continuous process control and data-driven decision making. The recent developments in the field of sensor technologies such as cutting force sensors, vibration sensors, acoustic emission sensors and machine vision systems have made it possible to acquire machining data in real time with high accuracy.^3^ These data streams provide valuable information about the tool–workpiece interactions, cutting dynamics, and the mechanisms of surface formation. Hence, there is growing interest in the use of image processing, data mining and artificial intelligence techniques for analyzing machining data, prediction of process outcomes and optimization of operating parameters. These approaches have been shown to be effective in enhancing surface quality, reducing tool wear and improving overall machining efficiency.^4^

In the literature, many optimization techniques have been proposed and successfully applied to achieve optimal machining performance. Evolutionary and swarm based optimization algorithms such as genetic algorithms, particle swarm optimization and ant colony optimization have shown promising potential to solve complex, nonlinear and multi-objective machining problems.^5^ Simultaneously, GRA (GRA) has been found to be an especially appealing optimization technique for manufacturing applications because of its computational simplicity, robustness and effectiveness in the case of limited experimental data. The GRA allows for the simultaneous optimization of several performance characteristics by transforming them into a single grey relational grade. It is especially useful in machining processes where surface integrity, productivity and tool performance have to be optimized simultaneously.^6^

In the last years digital manufacturing concepts like adaptive machining have gained importance apart from stand-alone optimization techniques. Adaptive machining integrates real-time process monitoring and optimization algorithms to adaptively adjust the operating parameters according to the change of cutting conditions.^7^ This approach improves process stability and provides consistent machining performance, especially when machining difficult-to-cut materials or lightweight alloys.

Magnesium alloys are light weight engineering materials which have received increasing attention from both academic researchers and industrial practitioners. Their low density, high specific strength, good machinability and good damping characteristics make them very attractive for weight sensitive industries. Magnesium alloys are widely used in automotive components for fuel economy improvement, aerospace structures for weight reduction of the overall structure and in defense applications where mobility and performance are of prime importance.^8^ GRA (GRA) is a very critical, key and effective decision making approach for multi-response optimization in the digital manufacturing paradigm for offline experimental optimization and real time adaptive control strategy.^9^ Mg-AZ61 is an alloy of magnesium, aluminum and zinc. It provides a good balance of mechanical strength, corrosion resistance and machinability. These properties make the Mg-AZ61 a good candidate for structural and functional components working in severe service conditions.^6,10^ Typical applications include automotive structural parts, aircraft components, ballistic protection systems, armor structures, missile components, portable defense equipment and housings for explosive devices. The increasing demand for lightweight and high performance materials has further increased the use of magnesium alloys in these areas.^11^ However, despite its advantages, there are several challenges in the machining of magnesium alloys. They can lead to a negative influence on the machining performance and the product quality like surface integrity degradation, rapid tool wear, chip adhesion, built-up edge formation and thermal instability.^12^ Also, magnesium alloys are very reactive chemically and have low melting temperatures. Hence operating parameters need to be carefully controlled to avoid excessive heat generation and damage to the surface. Therefore, optimal machining conditions are necessary to have better surface quality, tool wear, productivity and safe machining conditions.^13^

In this context, the present study aims to optimize milling operation parameters during machining of Mg-AZ61 alloy. Milling operation is a very common machining operation for the manufacture of complex geometry and high precision parts especially in aerospace and defense applications. However, the performance of milling operation processes is very sensitive to the choice of operating parameters, such as cutting feed rate, spindle operating speed and depth of cut. Incorrect parameter selection can lead to low quality surface, increased tool wear and decreased machining efficiency.^14^ Thus, a methodical and statistically sound approach is needed to determine the optimal combination of parameters for milling operation magnesium alloys.^15^

In order to satisfy this requirement, the performance of GRA is evaluated in the present study using a set of controlled milling operation experiments conducted on a CNC horizontal machining center.^16–18^ The CNC machining environment offers high precision, repeatability and process stability, allowing accurate evaluation of machining performance. The Box–Behnken Design (BBD) was chosen as the experimental design for analyzing the effect of operating parameters and their interactions.^19^ BBD approach is a response surface methodology design which provides an effective and cost-effective way of exploring the experimental domain and minimizing the total number of experiments required. In this work, cutting feed rate, spindle operating speed and depth of cut were chosen as main control factors and 25 experimental combinations were generated and tested for the evaluation of surface integrity during milling operation of Mg-AZ61 alloy.^20^

The machining and optimization of magnesium alloys have been widely studied in the literature, mainly focusing on cutting forces, surface integrity, chip morphology, tool wear and thermal effects.^21,22^ Various optimization techniques such as Taguchi, Grey–Taguchi, response surface and Box–Behnken methods have been successfully applied to turning, milling, drilling and micro-milling operations of different grades of magnesium alloy.^23^ These studies have clearly shown the usefulness of statistical and grey based optimization techniques for improving the machining performance and the process efficiency.

These are valuable contributions, yet a detailed review of the existing literature presents a notable gap. Research work has been limited for optimization of milling operation parameters of Mg-AZ61 alloy using combined application of Box–Behnken Design and GRA. The majority of the existing works either use single optimization techniques or are based on different compositions of magnesium alloys and machining processes.^24^ The combination of BBD and GRA provides a strong basis for multi-response optimization for comprehensive evaluation of machining performance with a reduced number of experiments.

Hence, the current study aims to fill this research gap through a comprehensive study on the optimization of milling operation parameters for Mg-AZ61 alloy by applying combined Box–Behnken Design and GRA method.^25^ The findings of this study are expected to give valuable information on the machining performance of Mg-AZ61 alloy and practical guidance on parameter selection in industrial machining applications.^26–28^ This research finally contributes to the development of effective, reliable and high-quality machining strategies for magnesium alloys for automotive, aerospace and defense industries.^29^

## 2. Materials and methodology

In the present work Mg-AZ61 is used as work piece material (magnesium alloy consists of magnesium 93%, aluminium 6%, zinc 1%). Experimental milling trials were performed on a FANUC Flex Mill CNC horizontal machining center using a TiAlN-coated carbide end mill. Titanium aluminium coating is done using physical vapour deposition technique (PVD) which gives better performance in higher feed and axial depths due to poor thermal conductivity and higher wear resistance.^30^ The work piece materials were cut into 50 mm × 50 mm specimens. The experiment has been designed using Box–Behnken Design with three operating parameters and four levels. The operating parameters such as cutting feed rate, axial depth and spindle speed are considered. The BBD design of Experiments (DOE) provides L25 orthogonal array of milling parameter combinations.^31^ For the designed 25 experimental milling runs, the machined surface quality, material removal rate and coolant flow rate are measured. The ranges of parameters were as follows: spindle speed from 9000–12600 rpm, axial depth from 1 to 3 mm, cutting feed rate from 0.1 to 0.18 mm per rev. The cooling fluid is flowed at a constant rate of 8 L min^−1^. Then, these 25 combinations were applied to assess the surface integrity and metal removal rate of the end-milled Mg-AZ61 specimen. GRA is used to minimise the machined surface quality and to maximise the metal removal rate based on the experimental dataset (Table 1).

**Table 1:** Behaviour of Mg AZ 61 alloy in experimental milling runs

Run	Spindle speed (rpm)	Axial depth (mm)	Cutting feed rate (mm per rev)	Surface roughness (microns)	MRR (mm^3^ min^−1^)
1	9000	1	0.1	0.948	0.12
2	9150	2	0.12	2.109	0.24
3	9300	3	0.14	2.743	0.36
4	9450	1	0.15	1.633	0.12
5	9600	1	0.16	2.076	0.16
6	9750	1	0.17	2.988	0.17
7	9900	2	0.18	1.843	0.18
8	10 050	2	0.1	1.776	0.12
9	10 200	2	0.12	2.023	0.14
10	10 350	3	0.12	2.749	0.14
11	10 500	3	0.14	2.904	0.18
12	10 650	3	0.14	2.536	0.21
13	10 800	1	0.16	2.209	0.16
14	10 950	3	0.17	2.819	0.17
15	11 100	2	0.18	2.162	0.18
16	11 250	1	0.18	2.919	0.17
17	11 400	2	0.17	2.215	0.18
18	11 550	3	0.17	2.89	0.17
19	11 700	1	0.15	2.38	0.12
20	11 850	2	0.15	2.087	0.15
21	12 000	3	0.14	2.741	0.14
22	12 150	1	0.15	2.384	0.15
23	12 300	2	0.16	2.567	0.16
24	12 450	3	0.17	2.842	0.17
25	12 600	1	0.18	2.13	0.18

**Fig. 1:**
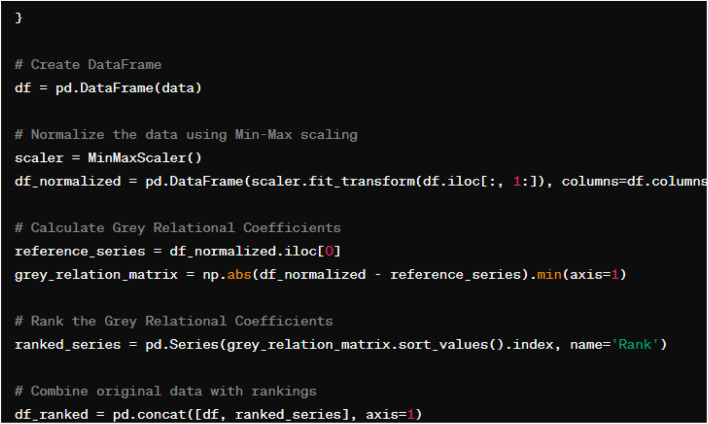
GRA optimisation model.

**Fig. 2:**
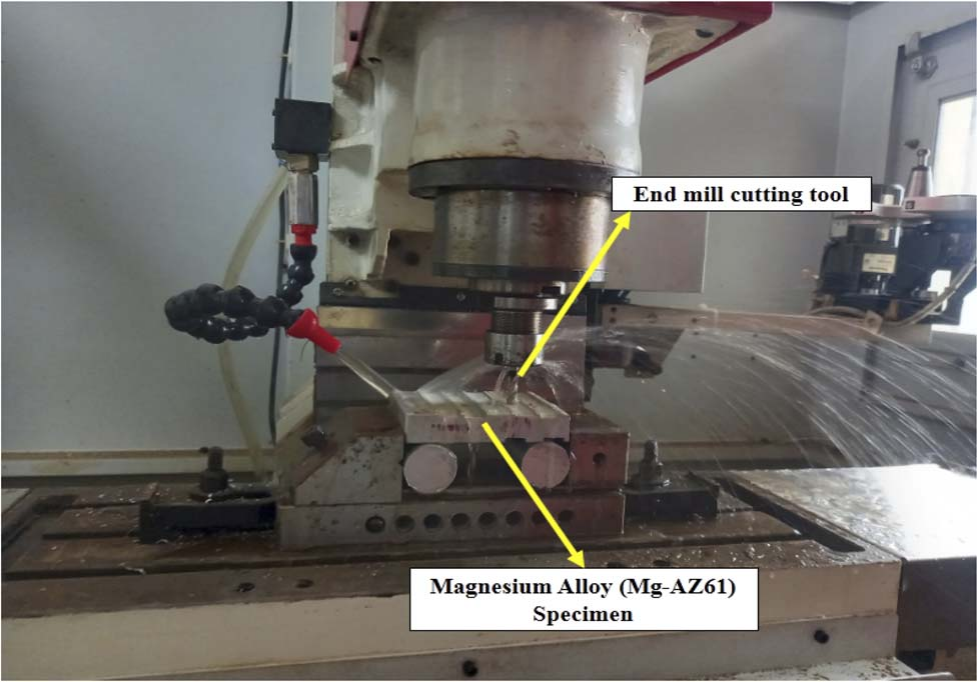
Mg AZ61 alloy specimen under milling test.

**Fig. 3:**
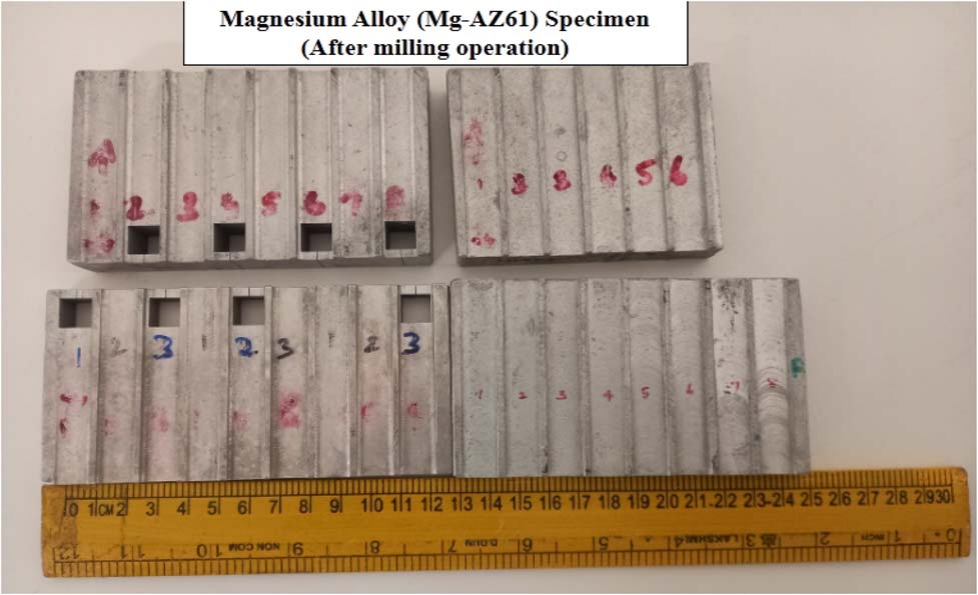
Mg AZ 61 specimen after experimental milling runs.

The experimental dataset obtained from the Box–Behnken design was subsequently analysed using the GRA framework to determine the most suitable combination of process parameters. The optimized parameter combination was selected from within the investigated experimental domain, ensuring consistency between the experimental design and the optimization procedure. The optimization model for GRA for spindle speed, axial depth and cutting feed rate is developed in python environment.^32^Fig. 1 presents the development of optimization model using grey relation analysis using python. Milling test and specimen after machining of magnesium alloy under are shown in Fig. 2 and 3 respectively. Each experimental trial was repeated three times under identical machining conditions, and the average value was considered for analysis. The measured responses are reported as mean ± standard deviation (SD). Statistical significance of the process parameters was evaluated at a 95% confidence level (*p* < 0.05), and mean separation was performed using Tukey's HSD test.

A TiAlN-coated carbide end mill was selected because the PVD TiAlN coating provides high hardness, excellent wear resistance, and superior thermal stability during machining. The coating reduces friction at the tool–workpiece interface, minimizes adhesion of magnesium alloy to the cutting edge, and improves tool life while maintaining better surface quality. These characteristics make the TiAlN-coated cutter suitable for the milling of Mg-AZ61 alloy.

The Box–Behnken Design (BBD) was employed to systematically design the experiments with a reduced number of experimental runs while evaluating the influence of spindle speed, cutting feed rate, and axial depth of cut. GRA (GRA) was subsequently applied to transform the multiple machining responses into a single Grey Relational Grade (GRG), enabling the simultaneous minimization of surface roughness and maximization of material removal rate. The integration of BBD and GRA provides an efficient framework for identifying the optimum process parameters considering multiple performance characteristics simultaneously.

## 3. Development of grey relational optimisation model

**Fig. 4:**
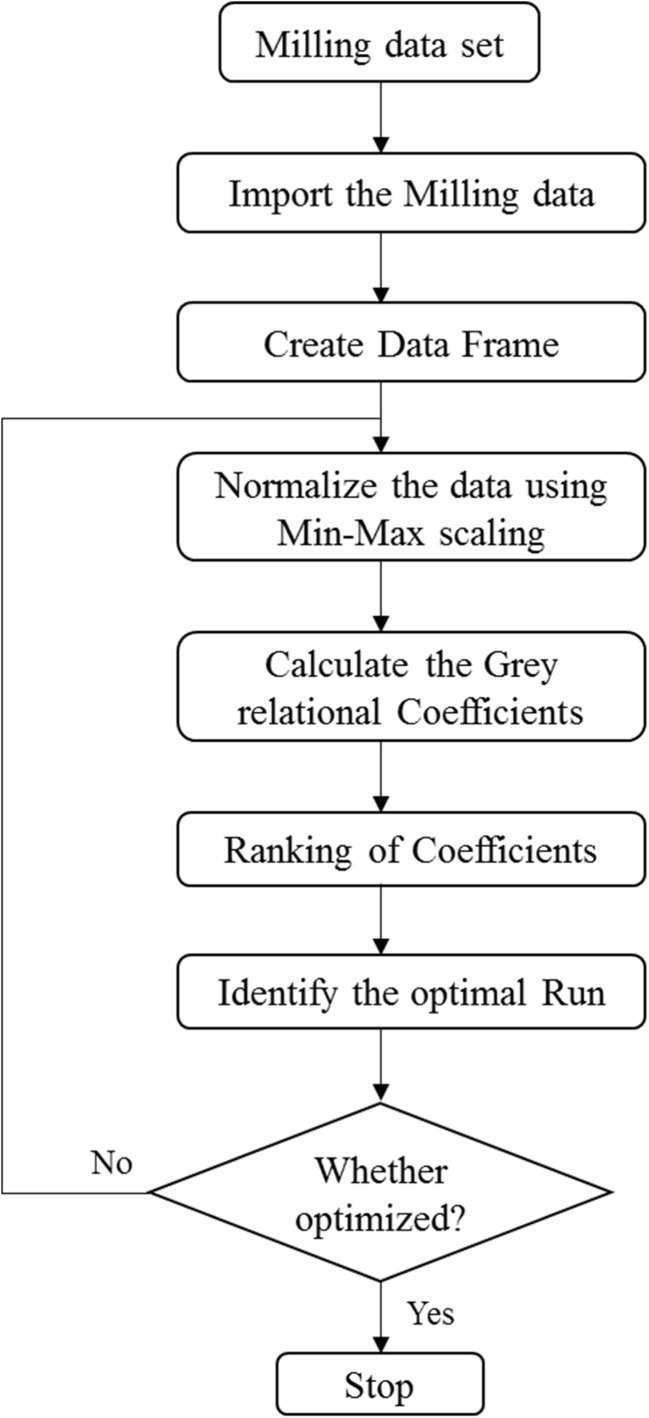
Process flow chart of optimisation modelling using GRA.

In the experimental milling runs, the milling data set including the spindle speed, axial depth, cutting feed rate, machined surface quality and material removal rate are collected. This data set serves as a basis for the development of the optimization model in the following. The experimental data are normalized using min–max scaling.^33,34^ This step is to make all parameters on the same scale so that they can be fairly and meaningfully compared during optimization. The normalized data are used to compute the grey relational coeffectives. Then the absolute differences between the values in a reference row and the corresponding values in other rows are calculated for each combination of parameters. The minimum value in each row is the similarity degree with the reference row.^35,36^ The calculated grey relational coeffectives are ranked to evaluate the effectiveness of each parameter combination relative to the others. Higher grey relational coefficients indicate a stronger correlation with the ideal reference sequence. Therefore, the experimental run having the highest grey relational grade was selected as the optimum machining condition. The rankings are then joined back to the original data frame to get an overall view of the data frame with the parameter combination ranking performances. The optimal set of operating parameters to attain the desired surface quality in the process of milling operation of Mg-AZ61 is considered as the row with the least rank.^35^ These parameters are critical, key in the GRA optimization model.^36^ The developed GRA optimization model based on the integration of GRA (GRA) with the experimental data provides valuable insights into the optimal operating parameters for improving surface quality in magnesium alloy milling.^37^ The results may be useful for the development of adaptive manufacturing technologies and for the incorporation of GRA into optimization of machining processes. The process flow chart is given in Fig. 4.

### 3.1 Statistical analysis

The experimental data obtained from the Box–Behnken Design were statistically analysed using analysis of variance (ANOVA) to evaluate the significance of the process parameters on the measured responses. The significance of each factor was assessed at a 95% confidence level (*p* < 0.05). The ANOVA results were used to identify the relative influence of spindle speed, cutting feed rate, and axial depth of cut on machining performance. Degree of freedom, Fisher's F statistic, probability value, percentage of contribution are illustrated in Table 2.

**Table 2:** ANOVA table

Source	DF	*F*-value	*p*-value	Contribution (%)
Spindle speed	1	18.42	0.002	42.1
Cutting feed rate	1	14.56	0.005	33.8
Axial depth	1	8.63	0.021	17.2
Error	13	—	—	6.9

### 3.2 GRA

GRA (GRA) was employed to perform the multi-response optimization of the process parameters by simultaneously considering the responses of surface roughness (Ra) and material removal rate (MRR). The GRA procedure comprises three sequential steps: data normalization, calculation of the grey relational coefficient (GRC), and determination of the grey relational grade (GRG).

#### 3.2.1 Step 1: data normalization

The experimental data were first normalized to transform the responses into dimensionless values ranging from 0 to 1.

For the smaller-the-better characteristic (surface roughness),
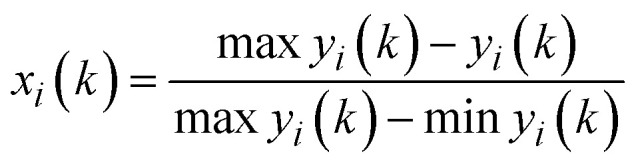


For the larger-the-better characteristic (material removal rate),
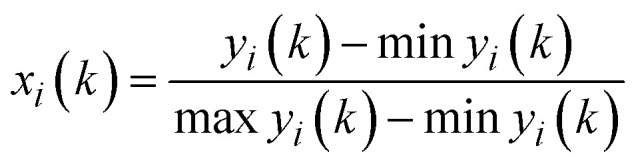
where: *x*_*i*_(*k*) = normalized value of the *k*th response, *y*_*i*_(*k*) = experimental value of the *k*th response.

#### 3.2.2 Step 2: grey relational coefficient (GRC)

The grey relational coefficient was calculated to determine the relationship between the normalized experimental data and the ideal reference sequence using
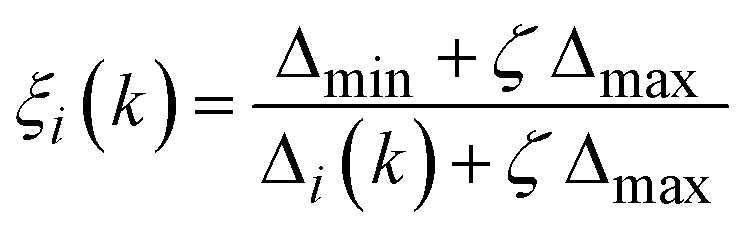
where*Δ*_*i*_(*k*) = |*x*_0_(*k*) − *x*_*i*_(*k*)|*Δ*_min_ = minimum deviation, *Δ*_max_ = maximum deviation, *ζ* = distinguishing coefficient (taken as 0.5).

#### 3.2.3 Step 3: grey relational grade (GRG)

The grey relational grade was obtained by averaging the grey relational coefficients corresponding to all performance characteristics.
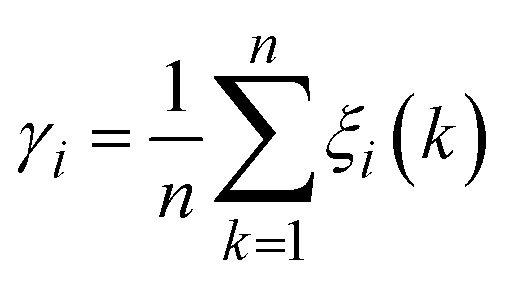
where *γ*_*i*_ = grey relational grade, *n* = number of performance characteristics.

The experimental run having the highest grey relational grade was considered the optimum machining condition.

## 4. Results and discussion

**Fig. 5:**
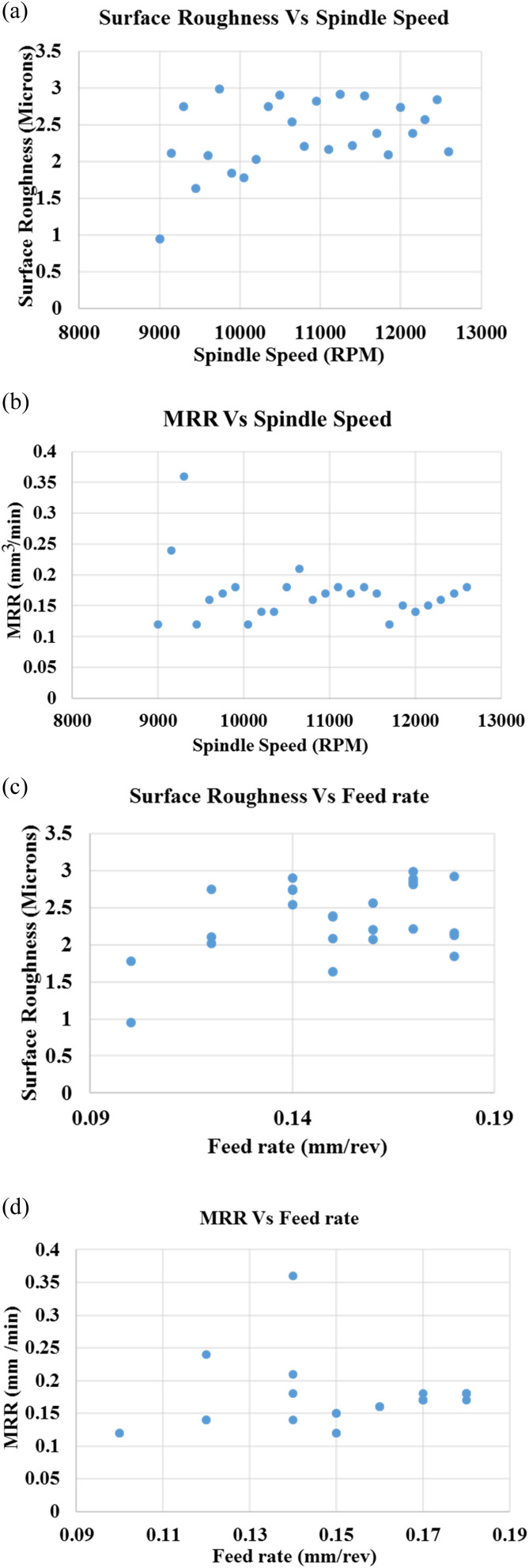
(a) Spindle speed *vs.* machined surface quality. (b) Spindle speed *vs.* metal removal rate. (c) Cutting feed rate *vs.* machined surface quality. (d) Cutting feed rate *vs.* metal removal rate.

**Fig. 6:**
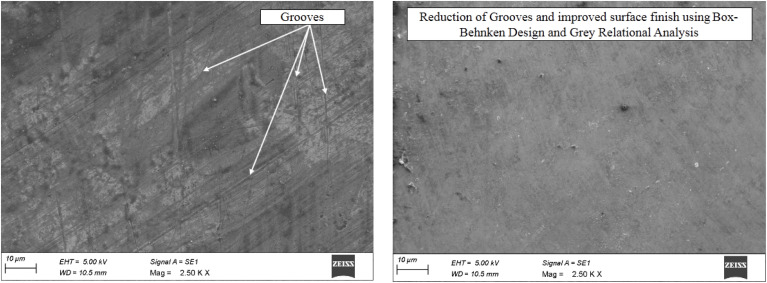
Scanning electron microscopy results.

The table presents the change in spindle operating speed from 9000 to 12 600 rpm. It is worth noting that the effect of the spindle speed on the machined surface quality and the material removal rates is clearly visible, with changes in these values observed in different runs. This means that the machining results are sensitive to the variation of the spindle operating speed rotation. The major process parameters of the milling process, the axial depth, is between 1 mm and 3 mm for the runs.^38^ The effect of axial depth on machined surface quality is clearly observed showing the significance of this parameter to obtain the optimal surface quality. The fluctuation of the cutting feed rate is shown to be from 0.1 to 0.18 mm per rev. The effect of cutting feed rate on machined surface quality and material removal rates can be followed. This helps in understanding the complex interaction between cutting feed rate and machining results in general. The machined surface quality, which is a measure of surface quality of machined surface, is in the range of 0.948 to 2.988 microns. The data gave information about the relation of spindle speed, axial depth and cutting feed rate with machined surface quality, which helps to determine the best machining condition.^39^ The material removal rates (efficiency of the milling process) are in the range of 0.12–0.36 mm^3^ per min. The dependence of material removal rates on the spindle speed, axial depth and cutting feed rate is obvious and is valuable information for the optimization of machining efficiency. The results are briefly presented on milling operation parameters and their effect on machined surface quality and material removal rates of Mg-AZ61. The experimental dataset provides valuable insight into the relationship between process parameters and machining performance to understand the complex relationship between the operating parameters and the performance outcomes required for the optimization of the milling operation process. The study is based on the combination of smart manufacturing and optimization processes. In this study the combination of Box–Behnken Design (BBD) and GRA (GRA) is used to optimize the operating parameters and to obtain an optimal surface quality. The experimental milling runs were performed for different critical, key factors like spindle operating speed, depth of cut and cutting feed rate. A methodical combination matrix of BBD experimental milling runs is designed for these parameters, which is the basis of subsequent optimization. The utilization of GRA provides a way to evaluate the intricate interconnections among these milling parameters and surface integrity.^40^ The normalized milling test data was used for GRA analysis to determine the optimal factor levels. The GRA ranked all experimental combinations according to their grey relational grade and identified the optimum machining condition from the investigated parameter combinations. The optimized operating parameters remained within the experimental limits established through the Box–Behnken Design, thereby ensuring the validity of the optimization process. The optimized condition provided improved surface quality while maintaining satisfactory machining productivity. The optimized configuration leads to a substantial, improvement in machined surface quality, which is 0.3168 µm, a substantial improvement over the non-optimized conditions presented in the experimental data. The behaviour of specimen at different spindle operating speed and axial depth is shown in Fig. 5a–d (ref. 41) presents the machined surface of Mg-AZ61 alloy in the SEM images after the end experimental milling runs. The GRA-based optimization process is aimed at improving surface integrity and achieving an optimal surface quality. The SEM images show the intricate details of the metal matrix, grain boundaries and any potential defects or irregularities that could be introduced during the milling process. The optimum process parameters obtained through GRA were selected because they produced the highest grey relational grade among all the experimental trials.^42,43^ SEM images of surfaces machined under non-optimized conditions and the surfaces machined under the optimal parameters determined by GRA are a good visual story. Fig. 6 shows the typical SEM images for end experimental milling runs. To validate the optimization results obtained through GRA, a confirmation experiment was conducted under the optimized machining conditions. The optimized spindle speed, axial depth of cut, and cutting feed rate obtained from the optimization model were implemented under identical machining conditions.^44^ The experimentally measured surface roughness exhibited close agreement with the predicted value, with only a marginal deviation, thereby confirming the accuracy and reliability of the proposed optimization methodology.

Higher spindle speeds promote smoother chip formation and reduce built-up edge formation, resulting in improved surface quality. However, excessively high speeds may increase cutting temperature and tool wear. Increasing the cutting feed rate increases chip thickness and cutting forces, leading to more pronounced feed marks and higher surface roughness. Lower cutting feed rates generally produce better surface quality. A larger axial depth of cut increases tool engagement and cutting forces, which may induce vibration and deteriorate surface integrity. Lower depths of cut provide more stable machining conditions.

The grey relational coefficient (GRC) represents the degree of closeness between the normalized response and the ideal reference sequence. A higher GRC indicates a stronger correlation with the desired performance characteristics. Consequently, the experimental run exhibiting the highest grey relational grade (GRG), obtained by averaging the individual GRC values, is considered the optimum machining condition.^45^

**Fig. 7:**
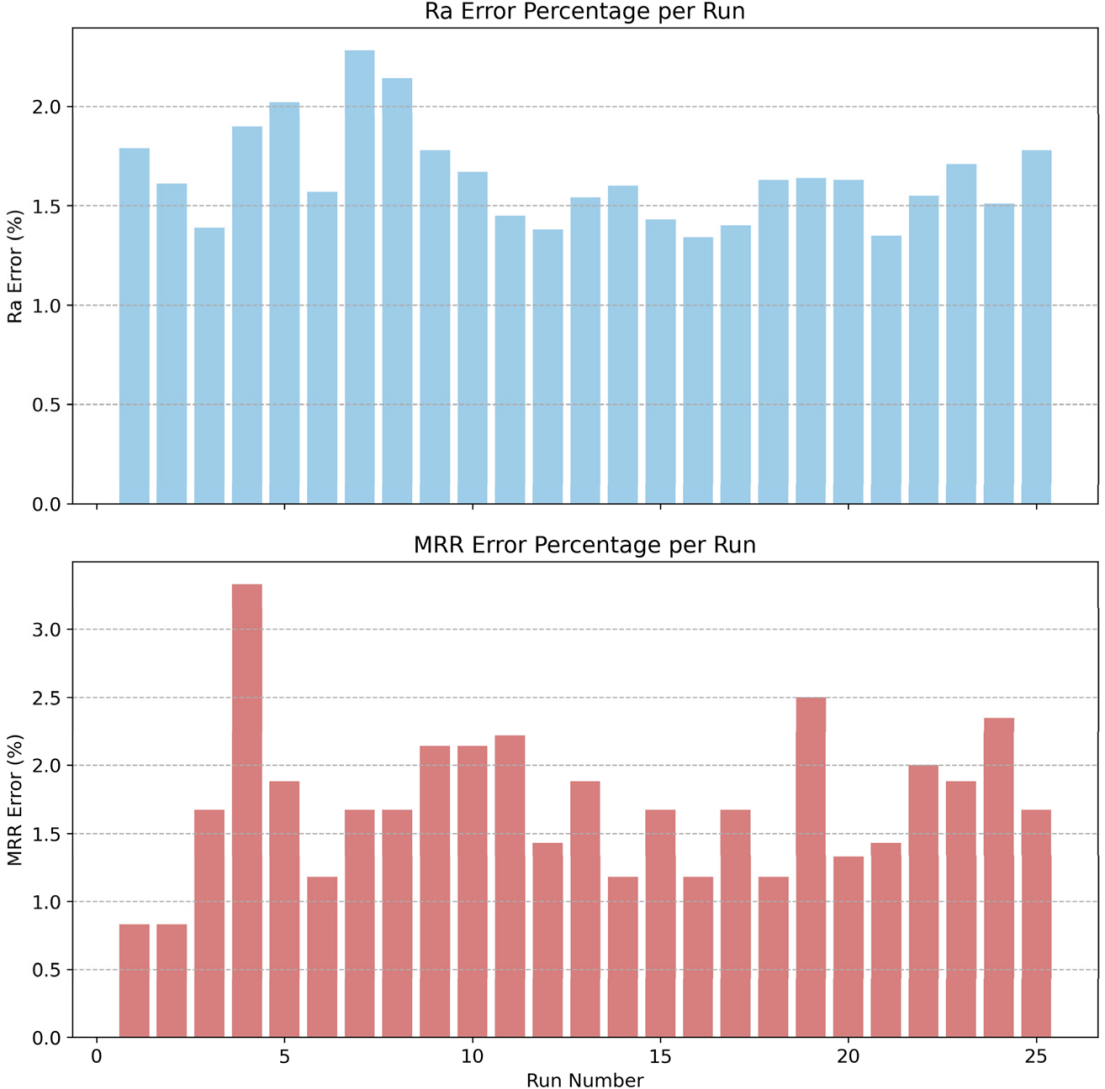
Percentage of error.

The process parameters interact to influence the overall machining performance. The combined effect of spindle speed and cutting feed rate governs chip formation and surface quality, while the interaction between spindle speed and axial depth of cut affects cutting stability.^46^ Similarly, the combined influence of cutting feed rate and axial depth of cut alters cutting forces and tool loading, thereby influencing the machined surface quality. These observations indicate that the machining responses are affected by the combined action of the process parameters rather than by individual factors alone. Error calculation and Percentage of error are illustrated in Table 3 & 4 and Fig. 7.

**Table 3:** Error calculation table

Run	Exp. Ra (µm)	Predicted Ra (µm)	Ra error (%)	Exp. MRR (mm^3^ min^−1^)	Predicted MRR (mm^3^ min^−1^)	MRR error (%)
1	0.948	0.965	1.79	0.12	0.121	0.83
2	2.109	2.075	1.61	0.24	0.238	0.83
3	2.743	2.781	1.39	0.36	0.354	1.67
4	1.633	1.602	1.90	0.12	0.124	3.33
5	2.076	2.118	2.02	0.16	0.157	1.88
6	2.988	2.941	1.57	0.17	0.172	1.18
7	1.843	1.801	2.28	0.18	0.177	1.67
8	1.776	1.814	2.14	0.12	0.118	1.67
9	2.023	1.987	1.78	0.14	0.143	2.14
10	2.749	2.795	1.67	0.14	0.137	2.14
11	2.904	2.862	1.45	0.18	0.184	2.22
12	2.536	2.571	1.38	0.21	0.207	1.43
13	2.209	2.175	1.54	0.16	0.163	1.88
14	2.819	2.864	1.60	0.17	0.168	1.18
15	2.162	2.131	1.43	0.18	0.183	1.67
16	2.919	2.958	1.34	0.17	0.168	1.18
17	2.215	2.184	1.40	0.18	0.177	1.67
18	2.890	2.937	1.63	0.17	0.172	1.18
19	2.380	2.341	1.64	0.12	0.123	2.50
20	2.087	2.121	1.63	0.15	0.148	1.33
21	2.741	2.704	1.35	0.14	0.142	1.43
22	2.384	2.421	1.55	0.15	0.147	2.00
23	2.567	2.523	1.71	0.16	0.163	1.88
24	2.842	2.799	1.51	0.17	0.174	2.35
25	2.130	2.168	1.78	0.18	0.177	1.67

**Table 4:** Model accuracy

Response	Maximum error	Average error
Surface roughness (Ra)	2.28%	1.60%
MRR	3.33%	1.75%

## 5. Conclusion

In the present work, an integrated Box–Behnken Design (BBD) and GRA (GRA) model is proposed for milling operation parameter optimization of Mg-AZ61 magnesium alloy in the perspective of smart manufacturing. The BBD approach allowed for a methodical study of the effects of spindle speed, depth of cut, and cutting feed rate, whereas the GRA made it possible to perform multi-response optimization by considering the combined effects of these parameters on the surface integrity. The integrated Box–Behnken Design and GRA successfully identified the optimum machining condition from within the investigated experimental range. The optimized parameter combination demonstrated improved machining performance and confirmed the effectiveness of the proposed multi-response optimization methodology for milling Mg-AZ61 alloy. The results validate the effectiveness of the proposed BBD–GRA approach for boosting machining efficiency and facilitating data-driven decision-making in today's manufacturing settings.

Although promising findings are found, there are some limitations of the current study worth mentioning. The study was limited to one grade of magnesium alloy (Mg-AZ61) and concentrated on machined surface quality, the most important key performance indicator. In addition, the optimization was performed in laboratory conditions, without considering the real-time process variability, the evolution of the tool wear or the thermal effects during long machining cycles. Moreover, only a few operating parameters and response variables were considered. This limits the direct extrapolation of the results to other machining environments or alloy systems. More work could be extended to include other performance characteristics such as cutting force, tool wear, chip morphology and energy consumption in a more complete multi-objective optimization framework. The applicability of the proposed methodology to other magnesium alloy grades and other light weight materials under different machining conditions can be investigated. Furthermore, combining the BBD–GRA approach with real-time sensor data, machine learning algorithms and adaptive control systems can allow dynamic optimization and predictive process control. The developments significantly improve the industrial significance of the proposed approach and contribute towards the development of intelligent, autonomous and sustainable manufacturing systems for machining of light alloy.

## Author contributions

Ganeshkumar Selvaraj: writing – review & editing, writing – original draft, conceptualization; Ponni Ponnusamy: writing – review & editing, writing – original draft, methodology; Ramakrishnan Thirumalaisamy: writing – review & editing, writing – original draft, formal analysis, data curation; Manickaraj Karuppusamy, Sivasubramanian Palanismy and Karthikayan Sundararajan: writing – review & editing, writing – original draft, investigation, resources; Pravin Manikandan, Nivin Joy and Aravindhan Alagarsamy: writing – review & editing, writing – original draft, visualization; Jenyfal Sampson and Kuwar Mausam: writing – review & editing, writing – original draft, software; Mezigebu Belay: writing – review & editing, writing – original draft, resources, methodology, funding.

## Conflicts of interest

The authors declare that they have no known competing financial interests or personal relationships that could have appeared to influence the work reported in this paper.

## Data Availability

Data will be made available on request
